# The influences of faith on illness representations and coping procedures of mental and cognitive health among aging Arab refugees: a qualitative study

**DOI:** 10.3389/fpsyt.2023.1083367

**Published:** 2023-05-08

**Authors:** Lana Bridi, Dahlia A. Kaki, Behnan Albahsahli, Dania Abu Baker, Xara Khan, Raghad Aljenabi, Nissma Bencheikh, Melody K. Schiaffino, Alison A. Moore, Tala Al-Rousan

**Affiliations:** ^1^School of Medicine, University of California, San Diego, San Diego, CA, United States; ^2^School of Medicine, University of California, San Francisco, San Francisco, CA, United States; ^3^Herbert Wertheim School of Public Health and Human Longevity Science, University of California, San Diego, San Diego, CA, United States; ^4^School of Social Work, San Diego State University, San Diego, CA, United States; ^5^School of Social Sciences, University of California, San Diego, San Diego, CA, United States; ^6^School of Public Health, San Diego State University, San Diego, CA, United States; ^7^Center for Health Equity, Education, and Research, UCSD Moores Cancer Center, San Diego, CA, United States

**Keywords:** dementia, religion, spirituality, Alzheimer's, Self-Regulation Model, Islam, Christianity

## Abstract

**Introduction:**

Refugees experience higher rates of mental illness such as depression and post-traumatic stress disorder (PTSD) which are documented risk factors for dementia. Faith and spiritual practices have been shown to play a significant role in patients' understanding and coping with illness, however, this field of study among refugee populations remains lacking. This study aims to address this literature gap by examining the role of faith on mental health and cognitive health among Arab refugees resettled in Arab and Western countries.

**Materials and methods:**

A total of 61 Arab refugees were recruited through ethnic community-based organizations in San Diego, California, United States (*N* = 29) and Amman, Jordan (*N* = 32). Participants were interviewed through in-depth, semi-structured interviews or focus groups. Interviews and focus groups were transcribed, translated, and coded using inductive thematic analysis and organized based on Leventhal's Self-Regulation Model.

**Results:**

Faith and spiritual practices significantly impact participants' illness perceptions and coping procedures regardless of resettlement country or gender. Several themes emerged: (1) participants believe in the interdependent relationship between mental and cognitive health. (2) There is a self-awareness of the impact of the refugee experience and trauma on participants' mental health problems, leading to a belief of increased personal risk for developing dementia. (3) Spiritual fatalism (belief that events are predetermined by God, fate, or destiny) greatly informs these perceptions of mental and cognitive health. (4) Participants acknowledge that practicing faith improves their mental and cognitive health, and many read scripture to prevent dementia. (5) Finally, spiritual gratitude and trust are important coping procedures that build resilience among participants.

**Conclusions:**

Faith and spirituality play an important role in shaping Arab refugees' illness representations and coping procedures of mental and cognitive health. Holistic public health and clinical interventions tailored to the spiritual needs of aging refugees and incorporating religion in prevention strategies are increasingly needed to improve the brain health and wellbeing of refugees.

## 1. Introduction

The rates of global forced displacement have been persistently increasing, doubling in the past decade alone to reach 89.3 million forcibly displaced persons in 2021 due to conflict, fear of persecution, violence, or human rights violations (1). Due to ongoing conflict and political unrest, the Middle East and North Africa have become the largest producers of refugees. Specifically, over 9 million Iraqis are displaced (2) and 13.5 million Syrians have been displaced, representing more than half of Syria's total population (3). With this unprecedented surge in forcibly displaced people, the health care systems of many host countries are struggling to accommodate the needs of refugees, particularly their mental health needs (4, 5). Moreover, host countries are specifically grappling with the task of caring for aging refugees (6). For example, the government of Jordan—a country hosting a large number of refugees from neighboring Arab countries—has explicitly identified care for older refugees to be a “significant challenge” (7).

Refugees already face many risk exposures for poor mental and cognitive health pre-migration, during the migratory trajectory, and post-resettlement (8, 9). Arab refugees—commonly defined as Arabic-speaking refugees from the Middle East and North Africa (MENA)—have particularly high rates of mental illness including anxiety, depression, and post-traumatic stress disorder (PTSD) (10–12), and these mental health burdens have been observed in both Arab and Western countries (13). While researchers and public health experts have recently been invested in Arab refugee public mental health, not many have integrated cognitive health into the discussions despite current literature demonstrating the important relationships between mental and cognitive health (14).

Emerging evidence is showing that Arab refugees from countries torn by war like Iraq and Syria are more likely to report worse cognitive function (15). Dementia is an example of a cognitive disease whose relationship to mental health has been well studied. There is strong evidence of the bidirectional relationships between PTSD (16–18) and depression with dementia (19). Refugees from low-middle income countries are disproportionately affected by mental disorders where prevalence rates of major depressive disorder could reach up to 47% (20); while it is possible that mental illness puts refugees at higher risk for developing dementia, no studies to date have investigated this hypothesis. Nonetheless, previous research has linked increased dementia risk to migration (21). For example, one recent European population study demonstrated an increased risk of dementia among migrants as compared to native Europeans, suggesting a link between migration history and cognitive aging (22). However, more research incorporating a life course perspective is needed, including following refugees over time to assess causality.

As mental ill-health is a risk factor for dementia and poor cognitive health, the treatment and prevention of mental disease among aging Arab refugees is of great importance to public mental health. Arab refugees face many barriers to accessing adequate and effective psychotherapeutic treatment, leading to disparities in mental health outcomes (23). Additionally, mental health and mental health care-seeking are widely stigmatized in the Arab culture (24), yet Arab refugees often turn to faith-based coping for their mental health (25). The benefits of spirituality and faith-based coping of disease are well documented in the literature (26–28) and faith has been specifically shown to promote preservation of self among individuals with dementia (29). Thus, understanding the utilization of spirituality as a tool to improve mental and cognitive wellbeing among Arab refugees can yield insightful information to be integrated in public mental health for this population.

However, research on the role of faith on mental and cognitive health among aging Arab refugees is lacking. As healthcare providers and public health experts have been placing increased importance on providing culturally concordant care, especially to minoritized communities such as refugees, it is imperative to understand how Arab refugees' perceptions and care-seeking for mental and cognitive health are shaped by faith (25, 30). To address this gap in the literature, this study aims to document aging Arab refugees' reliance on faith for their mental and cognitive health both in Western and Arab countries. Specifically, this qualitative study explores the role of faith in illness representations and coping procedures for mental and cognitive health.

## 2. Theoretical framework

The Leventhal Self-Regulation Model, also known as the Common Sense Model of Self-Regulation, is widely used to investigate the processes by which an individual develops an understanding and management of threats to their health (31, 32). This conceptual model suggests that when faced with a health threat, individuals respond both cognitively and emotionally, building illness representations with five attributes: illness identity, timeline, consequences, causes, and controllability (32). Following the development of an illness representation, individuals develop coping procedures to deal with the illness and finally proceed to appraise the efficacy of the coping strategies and their outcomes. Establishing illness representations and coping procedures accordingly is a dynamic, iterative, and cyclical process that allows the individual to arrive at the coping procedure which has the greatest success in managing their health threat.

Research on multiple types of conditions, including physical and psychiatric, has frequently applied the Self-Regulation Model to investigate the complexity of patients' understanding of their disease and, consequently, its management (33). Studies have demonstrated that dysfunctional mental illness perceptions are associated with poor adherence toward care regimens and adverse health outcomes (34). Conversely, other research suggests that illness perceptions can positively inform self-management of disease such as implementing lifestyle changes and care-seeking behaviors (35). Thus, it is evident that applying the Self-Regulation Model to this study has potential to yield important, applicable insights into the understanding and coping of mental and cognitive health of Arab refugees.

## 3. Materials and methods

### 3.1. Design

This study was an exploratory qualitative investigation into the role of faith on mental health and cognitive health among Arab refugees resettled in Arab or Western countries. Applied thematic analysis was used in this study because of its inductive procedure that presents participants' experiences as comprehensively and accurately as possible (36). This study was reported using the Standards for Reporting Qualitative Research (SRQR) (37).

San Diego, California (CA), United States (US) and Amman, Jordan are both large hubs for Arab refugees and thus chosen as research sites for this study. California has resettled the most refugees in the US (38). San Diego, CA is one of the largest US resettlement cities, were 21.5% of the population are immigrants and refugees (39), a majority of which are from Iraq and Syria (40). Overall, Jordan hosts an estimated 1.36 million Syrian refugees, 90% of whom resettled in urban areas such as Amman (41). Thus, these two research sites provide a strong representation of Arab refugees resettled in both Western and Arab countries.

### 3.2. Participants

Participants were Syrian and Iraqi refugees resettled in San Diego, California, US, and Syrian refugees resettled in Amman, Jordan. Inclusion criteria were: (1) having a present or former refugee status and (2) identifying as Arab race and/or born in the MENA region, and (3) being a native Arabic speaker. Exclusion criteria were: (1) anyone under 21 years old and (2) those unable to provide informed consent.

Recruitment for US-resettled participants started in December 2021 through a convenience sample from a federally qualified health center partner population. Potential participants were contacted *via* phone and advertising the study on social media platforms. Interested participants were screened for inclusion and exclusion criteria over the phone and consented. Participants were then scheduled for either virtual in-depth interviews or gender-concordant focus groups that occurred between March and August 2022. Recruitment for Jordan-resettled participants occurred in July 2022 through a convenience sample from an ethnic-based community organization partner population in the capital of Jordan, Amman. Recruitment was through random calling using a population of refugee beneficiaries obtained from the community organization. Similar to the recruitment strategy in the US, interested participants were screened, consented, and assigned to in-person, gender-concordant focus groups that occurred in July 2022. A total of 61 participants completed this study, 32 of whom were from Amman, Jordan and 29 of whom were from San Diego, CA, US.

### 3.3. Data collection

Data was collected by native speakers in Arabic through semi-structured, in-depth interviews and focus groups, and a questionnaire for demographic data. The interview/focus group guide was developed by vetted field experts and direct providers after conducting a scoping review of the literature (see Supplementary material). Interviews and focus groups explored perceptions of mental health, its relation to cognitive health, and behaviors to improve mental and cognitive health. To facilitate discussion on cognitive health, dementia was used as an example of cognitive ill-health. Interviews and focus groups ranged from 30 to 60 mins and were conducted by trained bilingual Arabic-speaking investigators either on a password-protected video-conferencing platform or in-person. In-person focus groups were conducted at volunteer participants' homes in Amman. All interviewers were trained by the research team on qualitative interviewing techniques and practiced with the interview guide before data collection to ensure proper technique. These techniques, such as probing participants and asking them to support their statements with examples, ensured prolonged engagement, a strategy enhancing the credibility of qualitative data (42). Interviews were audio-recorded, transcribed in Arabic, translated into English, and reviewed for content and accuracy.

### 3.4. Data analysis

Inductive thematic analysis was used to analyze the interview transcripts. To enhance the credibility of the analysis, investigator triangulation was implemented; four members of the research team independently reviewed and coded each interview transcript using ATLAS.ti software and met regularly to establish a codebook. Coders used the subjective assessment method to establish intercoder agreements (36). Analysis was done through the identification of recurrent themes following Crabtree and Miller's five-step interpretive process (43). Transcripts were analyzed until thematic saturation was reached.

### 3.5. Ethical approval

The Institutional Review Board (IRB) at the University of California, San Diego approved this research (IRB# 201634, 190483). Local approval for data collection in Jordan was approved through the community-based organization whose board is 30% Syrian refugees.

## 4. Results

### 4.1. Participant characteristics

Data on demographics and self-rated health and memory of participants is provided in Table 1. A total of 32 participants are resettled in Jordan and 29 participants are resettled in the US. The mean age of all participants is 57.6 years (SD 10.7) and the average length of stay in the country of resettlement is 7.8 years (SD 3.1). Participants originally from Syria accounted for 82.0% of participants with Iraq (14.8%) and Iran (3.2%) accounting for the remainder. Among participants, 34 (55.7%) were female and 54 (88.5%) were married. There was a range of levels of education with 41 (67.2%) having high school or a lesser degree as their highest level of education and 9 (14.8%) as illiterate. A total of 50 (82.0%) participants reported they were not employed.

**Table 1:** Participant demographics and self-rated health and memory.

	**Jordan**	**US**	**All**
	***N*** = **32**	***N*** = **29**	***N*** = **61**
**Gender**			
Female	17 (53.1)	17 (58.6)	34 (55.7)
Male	15 (46.9)	12 (41.4)	27 (44.3)
Age^*^ (mean, SD)	60.1 (5.1)	55.1 (14.1)	57.6 (10.7)
<50 years	–	8 (27.6)	8 (13.1)
50–59 years	20 (62.5)	6 (20.7)	26 (42.6)
60–69 years	9 (28.1)	12 (41.4)	21 (34.4)
>70 years	1 (3.1)	3 (10.3)	4 (6.6)
**Country of origin**			
Syria	32 (100)	18 (62.1)	50 (82.0)
Iraq	–	9 (31.0)	9 (14.8)
Iran	–	2 (6.9)	2 (3.2)
Years in resettled country (mean, SD)	8.9 (1.1)	6.6 (4.1)	7.8 (3.1)
**Highest level of education**			
Illiterate	6 (18.8)	3 (10.3)	9 (14.8)
Less than high school	20 (62.5)	6 (20.7)	26 (42.6)
High school	5 (15.6)	10 (34.5)	15 (24.6)
Undergraduate	1 (3.1)	8 (27.6)	9 (14.8)
Graduate	–	1 (3.4)	1 (1.6)
Vocational	–	1 (3.4)	1 (1.6)
**Employment status**			
Not employed	31 (96.9)	19 (65.5)	50 (82.0)
Employed	1 (3.1)	10 (34.5)	11 (18.0)
**Marital status**			
Married	28 (87.5)	26 (89.7)	54 (88.5)
Widowed	3 (9.4)	2 (6.9)	5 (8.2)
Never married	–	1 (3.4)	1 (1.6)
Separated	1 (3.1)	–	1 (1.6)
**Self-rated overall health**			
Poor	6 (18.8)	5 (17.2)	11 (18.0)
Not good	10 (31.3)	4 (13.8)	14 (23.0)
Average	16 (50.0)	9 (31.0)	25 (41.0)
Good	–	9 (31.0)	9 (14.8)
Excellent	–	2 (6.9)	2 (3.3)
**Self-rated memory**			
Poor	2 (6.3)	7 (24.1)	9 (14.8)
Not good	9 (28.1)	3 (10.3)	12 (19.7)
Average	10 (31.3)	9 (31.0)	19 (31.1)
Good	7 (21.9)	8 (27.6)	15 (24.6)
Excellent	4 (12.5)	2 (6.9)	6 (9.8)

* Percentage is <100.0 due to participants electing not to answer certain questions.

Participants were asked to rate their overall health and memory ranging from poor, not good, average, good, to excellent. In total, 25 (41.0%) participants reported poor or not good health while another 25 (41.0%) reported average health. Participants resettled in Jordan rated worse health with 50.0% reporting poor or not good health and 50.0% reporting average health. Self-rated memory was rated as poor or not good by 21 (34.4%) and good by 15 (24.6%). In contrast to self-rated health, participants resettled in Jordan rated their memory similarly to US-resettled participants.

**Figure 1:**
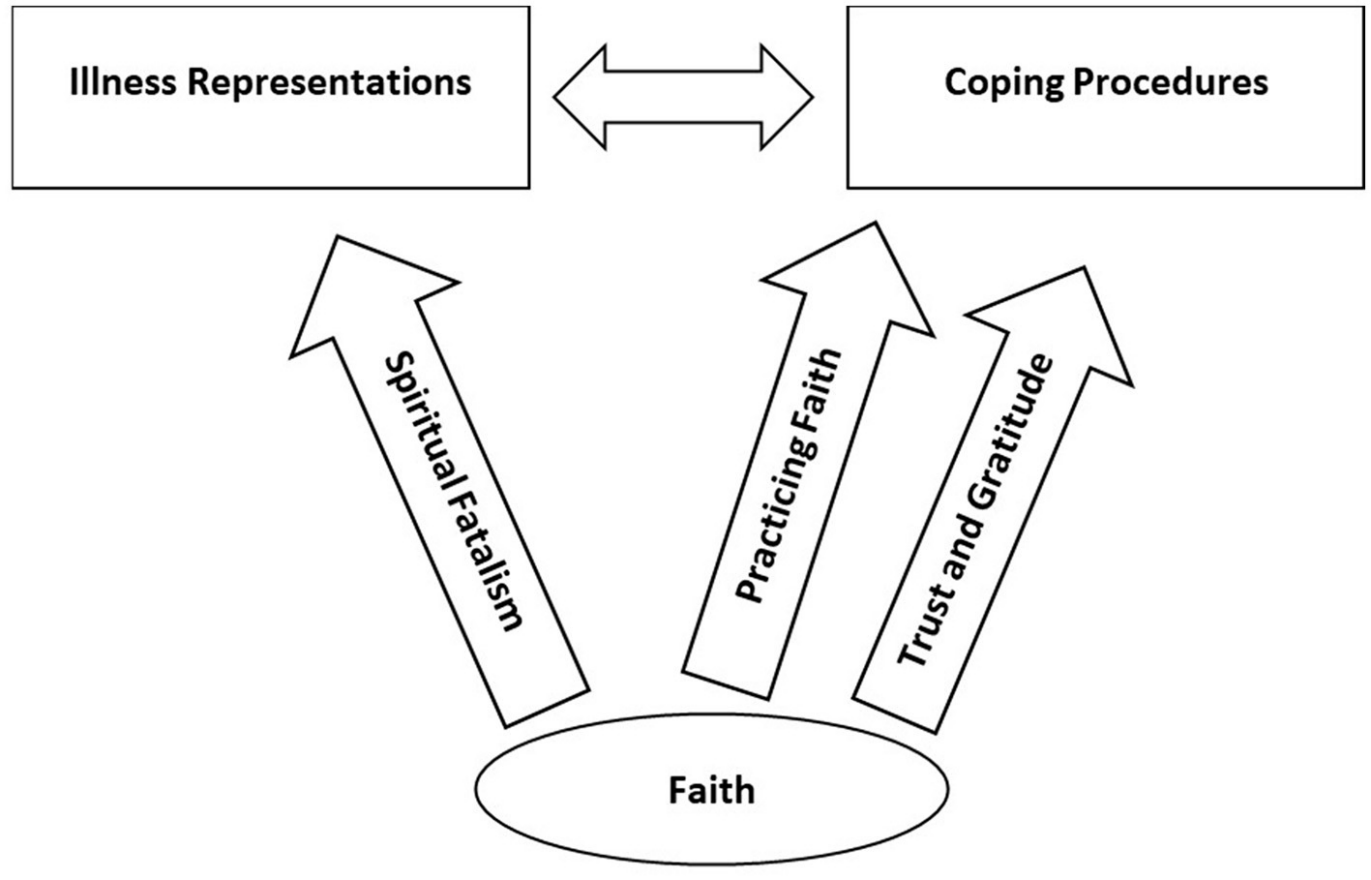
The influences of faith on aging Arab refugees' illness representations and coping procedures for mental and cognitive health.

The interviews and focus groups from this study yielded five themes on the illness representations and coping procedures of Arab refugees on mental and cognitive illness, and the impact of faith on these factors (Figure 1).

### 4.2. Illness representations

#### 4.2.1. Belief in the relationship between mental and cognitive health

A large majority of participants believed in a significant relationship between mental and cognitive health, specifically for dementia. Many believed that mental ill-health—caused by adverse or stressful life events—is an important, if not the most important, cause of dementia:

“*I think dementia is caused by all the life stressors that a person goes through while getting older. Sometimes because of life and what people are going through, a person gets stressed and worried, and people leave their families.” (Female, US)*.

Some participants specified chronic worry and/or overthinking as a cause of dementia. For example, this participant explained his reasoning behind overthinking as a cause of Alzheimer's:

“*People say overthinking is what makes someone's brain explode. It's like a phone, if you store a lot of stuff in it you would have to delete other information. The brain is the same.” (Male, Jordan)*.

Another participant shared a similar illness representation:

“*Of course [stress can cause dementia]. People started losing their minds because of overthinking. Because of that you either get a heart attack or your brain starts functioning differently.” (Male, Jordan)*.

Other participants noted how mental ill-health can even precipitate dementia at an earlier age than is typical:

“*A lot of the times Alzheimer's happens to old people usually around the age of 50 or 60. Of course, there are some cases where people who are between 20 and 40 have it. And this of course is because of mental health issues and the experiences they're living through or what is happening around them. Generally, Alzheimer's isn't necessarily only loss of memory, but it's also thinking about bad experiences someone went through.” (Female, US)*.

Similarly, many participants cited mental health illnesses, and experiences that negatively impact mental health such as trauma, as major risk factors for poor cognitive aging:

“*Trauma, wars, pain, depression, and fear. These are the major [dementia] risks of course.” (Male, Jordan)*.

Additionally, some participants highlighted how social isolation can impact both mental ill-health and dementia. Many believed that social isolation exacerbates risk factors for dementia. Conversely, participants believed that engaging in regular social activities can improve cognitive aging and prevent dementia. A few participants explained:

“*For example, if a person is 50 years old and he has kids but they're all married and he's just by himself in the house and they don't visit him, he just becomes isolated so this would affect the chances of getting Alzheimer's more.” (Female, US)*.

“*One shouldn't be by themselves. If one is by themselves, they will keep thinking about the past and depressing things and this will affect them negatively. And if one is closed off and quiet. I know that if one is social and talkative, they won't get Alzheimer's.” (Female, US)*.

While many believed that poor mental health negatively impacts cognitive aging, some participants believed the converse relationship to be true where positive mental health can prevent dementia. One participant who believed she had Alzheimer's explained this further:

“*How many years do I have left and I still haven't seen my children. But if I can see them it's going to affect my brain health positively and my Alzheimer's would not come because there's some brain activity and happiness. So of course, happiness and having fun with your loved ones helps.” (Female, US)*.

Similarly, another participant explained how, with the proper life conditions, one can achieve positive mental health and prevent dementia:

“*I think that the fear that people were living in affects their chance of having Alzheimer's in the future. But if the person is living in peace and they are active and have a settled life, then they wouldn't have exposure to this disease in my opinion.” (Female, US)*.

#### 4.2.2. Self-awareness of poor mental health and cognitive aging resulting from the refugee experience

Many participants shared how the refugee experience impacted their mental health and acknowledged how it resulted in their mental ill-health. Participants often compared their current mental health state to that before their refugee experience and noted a worsening in health. For most, trauma from their refugee experiences such as war, violence, and loss of socioeconomic status was the culprit in their deteriorating mental health:

“*All the things we've been going through, all the war, it's influencing us physically and mentally. I personally am in a special mental, societal, and psychological state. I changed a lot... I am struggling right now.” (Male, Jordan)*.

“*The experiences that I lived in Iraq from the heart and sadness and depression, believe me, you're going to laugh but every week once or twice I dream about it. I ask my daughter, ‘why do I dream about it?' and she says because it was a tough time and, until now, it's stuck with you. I dream about the war, the segregation, the depression, and everything.” (Female, US)*.

Others cited restrictive policies that prevented migration and family reunification as a source of mental ill-health:

“*My sister here, both of her sons traveled to America. They filed for family reunification but they are not accepting it in America. She sits and cries. Because of this she has diabetes, hypertension, and heart problems. Overthinking is causing disease and depression.” (Female, Jordan)*.

“*I always think about my children [who are separated from me] and I'm always sad. Every time I see something I always want to cry, I can't hold it in. I always feel distressed.” (Female, US)*.

As detailed in the previous theme, since many participants believed in the relationship between mental and cognitive health, they argued that their mental ill-health from their experience as refugees has deteriorated their cognitive health and placed them at an increased risk for dementia. Several participants mentioned feeling an impairment in their cognitive function including forgetfulness, reduced executive function, and an inability to focus:

“*[Dementia] happens because of the overthinking and every time they say how did this happen? What are we going to do? What happened to us in Syria? How did we flee like this? There are days people forget. There are days you put something somewhere and you forget where you put it because of how we aren't in our right minds and as focused anymore.” (Female, US)*.

“*Personally, I noticed that about 5 to 6 years ago, my memory started to lessen and the reason behind it besides all the trouble we went through back home is unfortunately the long waiting times to hear back from immigration services about our interviews and possible travel times. When one feels trapped in a cage being told they can't leave yet, not anytime soon, it affects your memory and you can't function properly.” (Male, US)*.

Others specified how experiencing trauma as a refugee and avoiding remembering trauma places them at an increased risk for dementia:

“*There are things that we don't want to ever mention, like these things are really hard and we don't want to remember them. This would lead to Alzheimer's because you don't want to remember.” (Female, US)*.

“*I experienced abduction and had nightmares after, my psychological state was in distress. I suspect with more than 80% confidence that this had affected my memory. Because [years later], my son who was standing outside the home when they kidnapped me told me, Dad you were screaming really loud.' I don't remember screaming at all... My son tells me, ‘Dad, your voice was reaching the streets.' I think those painful experiences have a direct impact [on my cognitive health] in addition to deteriorating my brain cells.” (Male, US)*.

Some participants highlighted how refugees experience social isolation which can negatively impact their cognitive health and place them at increased risk for dementia:

“*A lot of refugees have traumatic experiences and went through oppression, so they are scared to go outside and function normally. This makes sense that they just stay at home and become isolated which will probably affect their chances of getting Alzheimer's.” (Female, US)*.

Overall, many participants felt their cognitive age declining at a faster rate than is expected due to their mental ill-health as refugees. Many compared their current cognitive function to those of their parents or grandparents, noticing how their relatives had better memory even at an older age. One participant summarized this sentiment:

“*We are aging faster before our time has come! It's the stress and worries. The worry made us age. Here, we feel one year equates to 4–5 years from when we were living in our home country.” (Female, Jordan)*.

#### 4.2.3. Spiritual fatalism impacts understanding of mental and cognitive health

When explaining their mental and cognitive illness representations, many participants referred to their belief in spiritual fatalism as an important influence:

“*I believe that [life] is all a learning curve. It raises as you are growing then it reaches a certain peak then starts going down. That's in God's hands and we all will die one day. We can't really stop that. Your relationship with God will not slow this [disease] down because it's all about what you are building in the second life after death, not your current life.” (Male, US)*.

This belief in spiritual fatalism offered a simple method to understand mental and cognitive health among participants. Moreover, spiritual fatalism was used by participants as a reason to explain why they are not invested in thinking about their mental and cognitive health. For example, when probed if their refugee experience affects their mental or cognitive health one participant mentioned:

“*I don't think about this and don't want to think about it, I always say whatever comes from God is beautiful.” (Female, US)*.

Similarly, when participants were asked how often they think of their cognitive health and/or developing dementia, one participant answered:

“*I'm one of those people who doesn't think about it. No, because we gave ourselves to God.” (Female, Jordan)*.

The belief in spiritual fatalism also impacted participants' perceptions of mental disease and dementia prevention. Many referenced spiritual fatalism when they mentioned that there were no behaviors that they could engage in to protect themselves from developing dementia:

“*It's all in God's hands. You never know what God has written for human beings. A person cannot protect himself. This is fate, and it's written.” (Female, Jordan)*.

“*I will leave it to God. May God not let me get to this stage [dementia] where it gets really serious.” (Female, US)*.

Moreover, belief in spiritual fatalism even served as a barrier to care-seeking behavior. For example, when one participant was asked what they can do to improve their mental and cognitive health, they explained:

“*My back hurts. I have done 3 surgeries in my knees and I can't walk, I get tired right away. The doctors told me that I need to get back surgery, but I got scared and I did not do it. I'm just leaving it to God... I never think about [preventing disease]. I leave everything to God.” (Female, US)*.

### 4.3. Coping procedures

#### 4.3.1. Practicing faith improves mental and cognitive health

Many participants cited faith and spirituality practices as important coping procedures for their mental health distresses. Participants mentioned how praying, reading scripture, and even hearing the Islamic call to prayer (Athan) can improve their mental health, specifically mentioning a calming effect elicited by these spiritual practices:

“*Me and my siblings go to the mosque together and we hope to God that nothing [bad] will happen. We do this and it is the best thing [to help our mental health].” (Male, Jordan)*.

“*If you are having a headache, read a verse [of the Quran], then you will feel comfortable. If you have a heavy weight on your chest, you feel comfortable. Not only are you reading, but also you are enlightened.” (Female, Jordan)*.

Moreover, many participants believed that their spiritual and faith-based practices serve as a protective factor from developing dementia and mental health disease. Participants explained that engaging in spiritual practices can increase relaxation and improve self-contentment:

“*Yes, [a religious person has a decreased risk of dementia]. The person relaxes. Like I've read a lot from the Bible and the stories and then I go to another world. The person becomes satisfied, and content and they don't want anything from this world. The path to religion is an important factor [at preventing dementia]. Sadly, here there's so much going on here and there so no one has the time. We have to give religion time because it's an important thing.” (Female, US)*.

Others referred to their spiritual practices as meditative experiences that can help clear the mind:

“*100% I believe faith can [prevent mental disorders or dementia]. When I pray, I am submitting myself to God. The same happens when I read the Quran, I am fully engaged. This clears up my mind. I put off all thoughts about the past. Praying is very important and that relationship with God is what you are left with at the end of the day.” (Male, US)*.

Moreover, one participant even linked a decline in faith and faith-based practices with increased prevalence of cognitive disease:

“*I've heard that if you read the Quran, or whatever religion that you're from, and pray and always think about God, the chance of getting Alzheimer's would be really low. Like in my family, everyone lived to 100 years old and they didn't have Alzheimer's. It seems that whenever science is advancing, this idea of Alzheimer's is increasing. Not to be rude, but it seems that people are deviating away from religion. Social life and exercise are important but, in my opinion, religion is the most important.” (Female, US)*.

Some participants noted how reading scripture improves their cognitive health and protects them from dementia:

“*I am helping myself by reading the Quran and reading books. This helps me keep my memory very strong. And I feel connected.” (Male, US)*.

“*I think that when someone continues reading books and continues learning and having knowledge and keeping your mind active is really important. So, if a person continues to read and memorize the Quran will not have Alzheimer's.” (Female, US)*.

A few participants shared how, when seeking care for their mental disorders, their mental health providers incorporated faith-based strategies into their care regimens. Participants acknowledged the efficacy and their appreciation of those strategies:

“*[My doctor] used to talk about brain and mental health because she knows about how Iraq had a lot of traumatic experiences. She said to speak about whatever I wanted, so I told her about everything. Honestly, I used to be scared to drive cars but she helped me. She said, ‘God is sitting with you and you aren't alone.' She changed me so much.” (Female, US)*.

“*I went to a doctor in Syria when I went through a lot of experiences. I told them I was depressed and I needed a therapist. And the therapist would say, ‘read Quran and pray two times.' And what she said is still with me. Religious connection is really important because God is helping you but you just have to be dependent on him. You have to be truthful to yourself, you have to focus.” (Female, US)*.

Similarly, another participant mentioned how they supplemented their mental health provider's care with faith-based practices to improve their mental health outcomes:

“*I came to [Jordan] and my son was trapped. I became depressed and I went to a mental health doctor. She helped me get better and I started to read the Quran and memorize verses which also helped me a lot.” (Female, Jordan)*.

Furthermore, one participant explained how spiritual coping procedures for mental ill-health are more important than pharmacological interventions:

“*I went to psychologist, like a therapist because I was not normal because my son was dead and I couldn't even just sit with myself. The doctor would give me pills so that it could help me but the pills don't help. The solution is in the soul, and you have to fix it yourself.” (Female, US)*.

#### 4.3.2. Spiritual gratitude and trust builds resilience

Participants often cited their spiritual relationships and expectations as an important coping procedure for their mental ill-health. Some mentioned how their gratitude for their situations, including their hardships and trauma, helps alleviate their mental ill-health:

“*Everyone has their struggles and issues. Every person—the place they come from, the land they're coming from, whether it's here or Iraq, everyone has their own life and issues. My daughter takes us out a little like to the mall when she goes shopping and that's it. We sit at home and the TV is on, and that's it. And I thank the lord for it.” (Female, US)*.

“*In this country, we keep crying about our situation but we thank God. We talk about how we don't get enough support [in Jordan] but we thank God we were able to come here. At least it's safe. It feels like we came to our people, thank God.” (Male, Jordan)*.

For others, trust and submission that spiritual protection and aid will be provided offer ease:

“*Everyone says, ‘how can you go through this and still survive? You've been here for how many years and you still haven't seen your children, how have you persevered?' I ask for God to help us. We depend on him.” (Female, US)*.

“*To be honest, sometimes because of how much I think I feel, I cannot think, and my body is tired. Sometimes I tell God I give all this to you in which I feel like that helps with a lot of my worrying.” (Female, Jordan)*.

“*Of course God didn't leave us. With hardship comes ease as God said. This is guaranteed.” (Female, US)*.

One participant mentioned how this trust will protect her and other refugees from developing dementia:

“*Our experience as refugees and in the camps will not lead to Alzheimer's because God helped us forget our past and we started a new life. Thanks to God we have trust in God that this all happened for a reason and everything that will happen in the future. We have trust in God that this was written for us.” (Female, US)*.

Some participants also shared how they encourage others to have similar spiritual relationships and trust to ease their mental health burdens:

“*My son has very bad mental health. Even though I am just as mentally tired, I still try to show him I am strong. I try to encourage him saying God will solve everything. God will solve this for us, do not worry about it.” (Female, Jordan)*.

## 5. Discussion

This study qualitatively examined the influences of faith on mental and cognitive health illness representations and coping procedures among Arab refugees who have resettled in Western or Arab countries, employing the Leventhal Self-Regulation Model. Data analysis yielded five pertinent themes. (1) Participants' illness representations were based on a belief in an inextricable link between mental and cognitive health. Mental ill-health was attributed as a major cause or risk factor for the development of dementia. (2) Participants also acknowledge their own mental ill-health as a result of the accumulation of traumatic refugee experiences and stress, showcasing the chronicity of the illness representation timeline. Due to participants' self-reported poor mental health, many believed in an increased personal risk of developing dementia—or having already developed the disease—based on the consequences of increased forgetfulness and lack of cognitive acuity. (3) Spiritual fatalism was found to be a significant influence on participants' illness representation of the controllability of mental health disease and dementia. Faith was an important coping procedure for participants in two major domains: (4) Engaging in faith-based practices improved mental and cognitive health by calming and increasing self-contentment and were believed to protect from dementia. (5) Believing in spiritual gratitude and trust in God as a protector developed resiliency. It is important to note that the themes identified from this study were consistent among participants regardless of whether participants resettled in a Western or Arab country. Similarly, themes were consistent across genders. The presence of strong faith influences among the diversity of the Arab refugee experience underscores the importance of faith in shaping illness representations and coping procedures within this group.

Current dementia literature supports participants' illness representation that their mental health and trauma experiences impact their risk for dementia. A recent meta-analysis quantified the association of PTSD and dementia risk and showed that PTSD is a strong risk factor for all-cause dementia (18). Additionally, a prospective cohort study showcased increasing depressive symptoms are a risk factor dementia (19). While the literature on dementia risk and cognitive decline among Arab refugees is severely lacking, a study of Palestinian children found that non-refugee children outperformed refugee children in sustained attention, verbal memory, and visual memory (44). It is evident from the results of this study that many participants reported depressive symptoms and/or described PTSD symptoms. Additionally, many participants reported deteriorating cognitive function and early aging; this trend is also exhibited in participants' self-rated memory as 34.4% reported poor or not good memory. These data trends highlight the need for increased research efforts into the relation between mental and cognitive health among Arab refugees. This area is particularly important given recent data published from a 30-year observational study finding people with early-life mental disorders to be at elevated risk of subsequent dementia and younger dementia onset (45). Implications of this study highlight the importance of integrating dementia prevention in mental disorder treatment, even at younger ages, and continuing future research to interrogate the mechanisms linking mental illness with dementia (45). Future efforts addressing these implications should include refugee populations. For example, studies quantifying depression and PTSD symptoms and examining associations with cognitive function among Arab refugees are warranted.

An important theme on spiritual fatalism influencing illness representations emerged from this study. Participants described how their belief in providential destiny regarding their health impacts their representations on controllability of mental and cognitive diseases such as dementia. This is an important finding because it demonstrates how a belief in spiritual fatalism can serve as a barrier to care-seeking behavior and/or engaging in disease prevention for this group, both of which are consistent with previous research. One qualitative study revealed that patients with lung cancer felt safe without seeking treatment due to their fatalistic belief (46). Similar results have been demonstrated specifically within Arab populations: a study among Jordanian- and Palestinian-American women found that fatalism and traditional healers consultations served as barriers to participation in breast cancer screening (47). However, a more recent study from Shahid et al. stratified fatalism into active and passive forms, where active fatalism is the belief that one must actively work to bring a predestined future into fruition. After distinguishing between these two forms of fatalism, they found that active fatalism was positively correlated with positive coping skills and negatively correlated with depression and external locus of control (48). These results are promising as therapies for mental health can be designed to shift a patient's classical view of fatalism to an active one to enhance cognitive control and improve disease prevention behaviors. This approach, in theory, would be more feasible than therapies attempting to remove fatalistic beliefs altogether. Furthermore, implementing community-based strategies to partner with local religious institutions and leaders to promote active fatalism in their ideology may be an important public health strategy for the aging Arab refugee community, especially given that belief in fatalism tends to increase with age (49).

Consistent with previous literature, this study found that practicing faith and believing in God's protection are important coping procedures for Arab refugees. A review from the United Nations High Commissioner for Refugees found that many Syrian refugees cope with psychosocial distress through praying (50). Another study from the United Kingdom demonstrated that Syrian refugees face several barriers to accessing mental healthcare services—including stigmatization of mental ill-health—and therefore turn to faith for support (51). However, our study is the first to demonstrate faith-based practices as coping procedures for both mental and cognitive health. Participants reported reading and reciting scripture as a method to calm and clear the mind, in addition to maintaining cognitive activity and improving memory. Lifelong cognitive enhancing activity, which most studies measure by proxy of education level, may delay the onset of dementia (52). Thus, it is important to note that faith-based practices can maintain cognitive enhancing activity among Arab refugees regardless of educational level. Furthermore, Arab refugees tend to have poor education statuses (53), including this study's participants-−67.2% achieving high school or less and 14.8% are illiterate—therefore relying on faith-based practices, such as memorizing scripture, to maintain cognitive activity may be an accessible and culturally concordant public health intervention to prevent dementia among Arab refugees.

A subtheme on the integration of faith-based coping procedures with professional mental healthcare emerged from this study. Participants who received faith-based mental healthcare endorsed its efficacy, accessibility, and feasibility. Additionally, a few participants took the initiative to self-integrate faith-based strategies into their mental healthcare regimen from their providers. These findings highlight the potential for faith-based health promotion to improve the mental health of Arab refugees. Such strategies include training mental health providers regarding cultural and religious backgrounds of refugee patients (25), training religious community leaders to refer refugees to mental health services (54), and mediating conversations between medical providers with experience and training in complementary and alternative medicine, mental health providers, and refugee patients (55). Incorporating faith-based health promotion strategies are starting to gain traction, especially for minoritized communities facing several barriers to healthcare (56, 57). For example, a community intervention developed by Chaudhary et al. for Syrian refugees in the US implemented a peer-to-peer healthcare training program at a local mosque. They found that over 2 years following the intervention, health educators at mosques significantly impacted the ability of refugees to assimilate to the US healthcare system, especially for services around mental health management (58).

Incorporating faith-based mental and cognitive health promotion may be a widely useful tool for the health promotion of aging Arab refugees both in Western countries and Arab countries. Lack of cultural competence and safety is a common barrier for minoritized communities to accessing healthcare in the West (30, 59). While issues with culturally concordant care might not be as prevalent for Arab refugees resettled in Arab countries, refugees continue to face many barriers due to poor healthcare infrastructures and distribution of resources (60, 61). Incorporating community and faith-based interventions can reduce these respective barriers to healthcare access. For example, faith-based interventions have been effective in shaping care-seeking attitudes for Muslim women to increase cancer screening uptake (62, 63). A randomized controlled trial from the National Cancer Institute found a significant effect favoring the intervention group—receiving immediate 15-month physical activity and healthy eating activities at African-American churches—in self-reported physical activity (64). Such evidence-based, community-partnered interventions have been well-studied in the literature for minoritized communities such as African Americans and Latinos (64–66) and strategies should be tailored to the aging Arab refugee population. Furthermore, a specific barrier to mental health treatment is the stigmatization of mental health disease, an issue that is prevalent among the Arab refugee community (67). Faith-based community interventions have been proposed to reduce stigma of mental illness (68). Further research into the efficacy and feasibility of such interventions is warranted.

## 6. Strengths and limitations

To the best of the authors' knowledge, this is the first study to investigate the influences of faith on illness representations and coping procedures of mental and cognitive health among aging Arab refugees. The qualitative exploratory approach used applied thematic analysis techniques in order to center the participants' perspectives in this study. Additionally, the diversity of the sociodemographic characteristics of participants, including the comparison between Western country and Arab country resettlement provides novelty to this study. While this research has many strengths, it is important to recognize that faith or religion is a sensitive topic, especially for Arab refugees who might have faced religious persecution or discrimination post-resettlement. Therefore, there is potential social acceptability bias in the results of the study since participants might have felt varying levels of comfort sharing their faith and faith-based practices to the researcher collecting data depending on whether they share a similar religious background or not. However, interviewers were Arabs of Syrian or Iraqi ancestry and trained to make efforts to ensure participants' comfort in sharing their opinions, for example, by stressing the lack of judgment. Both community-based organizations in the US and Jordan were secular entities which might have earned the trust of participants to speak more openly about their faith. Additionally, based on the sensitivity of religious topics and beliefs (sects such as Sunni and Shi'a Muslims) and historical mistrust of research by the Arab refugee community, we ought to not collect data on religious affiliation in the demographic information. However, based on the results from the interview, participants represented both Muslim and Christian sects popular in Iraq and Syria which is representative of the population in the MENA region. Another limitation is potential selection bias because, while the partnered organizations were good avenues to connect with local refugees, it is possible to have missed community-dwelling refugees who do not receive services from these organizations which may limit generalizability. However, we believe that San Diego and Jordan being large refugee hubs (38–41) gives a good representation of Arab refugees elsewhere.

## 7. Conclusions

The findings of the current study indicate that faith holds an important role in the illness representations and coping procedures of aging Arab refugees regarding mental and cognitive health. Arab refugees' faith offers both obstacles (i.e., spiritual fatalism) and resources (i.e., improvement in mental health, dementia prevention, and resiliency) to mental and cognitive health. The results confirm the importance and potential of implementing culturally concordant and faith-based mental and cognitive public health initiatives in Western and Arab host countries for Arab refugees. Additionally, the outcomes of this research could be beneficial for healthcare providers to leverage Arab refugee patients' faith-based practices to improve care-seeking behaviors and disease prevention. Future studies should investigate faith-based public mental and cognitive health interventions to improve health outcomes for the aging Arab refugee population.

## Data availability statement

The raw data supporting the conclusions of this article will be made available by the authors, without undue reservation.

## Ethics statement

The studies involving human participants were reviewed and approved by the Institutional Review Board (IRB) at the University of California, San Diego approved this research (IRB# 201634, 190483). The patients/participants provided their written informed consent to participate in this study. Written informed consent was obtained from the individual(s) for the publication of any potentially identifiable images or data included in this article.

## Author contributions

TA-R, DK, and LB conceived the study. LB, DAB, BA, and TA-R collected data. LB, DK, BA, and DAB analyzed data. LB, XK, and RA drafted the manuscript. All authors critically reviewed and approved the final version of the manuscript.
