# Supplementary material for: The influences of faith on illness representations and coping procedures of mental and cognitive health among aging Arab refugees: a qualitative study
Source: Front Psychiatry. 2023 May 8;14:1083367. doi: 10.3389/fpsyt.2023.1083367 (PMC10200919; doi:10.3389/fpsyt.2023.1083367)
Supplement: Supplementary file 1 [file Table_1.DOCX]

Supplementary Material

**Supplementary Table.** In-depth interview guide questions selected for qualitative data analysis

| **Domain of Inquiry** | **Questions** |
| --- | --- |
| Mental and cognitive illness representations | What comes to mind when you think about dementia or Alzheimer’s disease?  What puts people at risk for getting dementia?  What worries you about cognitive disorders?  Thinking about your own history as a refugee, what is it about the refugee experience that can affect health (e.g., mental, cognitive)?  What do you believe is your likelihood of developing dementia?  Do you believe dementia can be prevented? If yes, how? |
| Mental and cognitive health coping procedures | What do you think you can do to reduce the risk of or prevent dementia?  How do you take care of your mental and cognitive health?  What motivates you to take care of your mental and cognitive health? |
